# Review of various NAMPT inhibitors for the treatment of cancer

**DOI:** 10.3389/fphar.2022.970553

**Published:** 2022-09-07

**Authors:** Yichen Wei, Haotian Xiang, Wenqiu Zhang

**Affiliations:** ^1^ West China School of Pharmacy, Sichuan University, Chengdu, China; ^2^ State Key Laboratory of Biotherapy and Cancer Center, Department of Respiratory and Critical Care Medicine, West China Hospital, Sichuan University, Chengdu, China; ^3^ Department of Ophthalmology, West China Hospital, Sichuan University, Chengdu, China

**Keywords:** nicotinamide phosphoribosyltransferase, individual inhibitors, pharmacological combinations, dual inhibitors, antibody-drug conjugates

## Abstract

Nicotinamide phosphoribosyltransferase (NAMPT) is a rate-limiting enzyme in the NAD salvage pathway of mammalian cells and is overexpressed in numerous types of cancers. These include breast cancer, ovarian cancer, prostate cancer, gastric cancer, colorectal cancer, glioma, and b-cell lymphoma. NAMPT is also known to impact the NAD and NADPH pool. Research has demonstrated that NAMPT can be inhibited. NAMPT inhibitors are diverse anticancer medicines with significant anti-tumor efficacy in *ex vivo* tumor models. A few notable NAMPT specific inhibitors which have been produced include FK866, CHS828, and OT-82. Despite encouraging preclinical evidence of the potential utility of NAMPT inhibitors in cancer models, early clinical trials have yielded only modest results, necessitating the adaptation of additional tactics to boost efficacy. This paper examines a number of cancer treatment methods which target NAMPT, including the usage of individual inhibitors, pharmacological combinations, dual inhibitors, and ADCs, all of which have demonstrated promising experimental or clinical results. We intend to contribute further ideas regarding the usage and development of NAMPT inhibitors in clinical therapy to advance the field of research on this intriguing target.

## 1 Introduction

One of the features of aggressive cancer is cellular metabolism reprogramming (Tennant et al., 2010; Loree and Kopetz, 2017). The regulation of these metabolic processes and the generation of adenosine triphosphate (ATP), which is constantly needed in both normal tissues and tumor cells, depends on nicotinamide adenine dinucleotide (NAD) and nicotinamide adenine dinucleotide phosphate (NADPH). In tumor cells, the half-life of NAD is only about 1 hour, so the synthesis of more NAD is necessary compensate for such rapid degradation, which in addition to the NAD required for ATP supply, makes their demand for this compound even greater (Rechsteiner et al., 1976; Schreiber et al., 2006). Based on these findings, it has been hypothesized that disrupting NAD homeostasis by interfering with NAD biosynthesis processes, hence lowering the NAD pool in cancer cells, could be a promising technique in cancer therapy.

The three principal mechanisms for NAD synthesis are the Preiss-Handler pathway, the *de novo* pathway, and the salvage pathway. In the *de novo* pathway, we use tryptophan as a substrate to synthesize NAD in the organism through a series of enzymatic reactions. Because they hardly have all enzymes necessary to convert tryptophan to NAD, several cancer cell lines have been shown that they cannot utilize the *de novo* pathway (Heyes et al., 1997; Xiao et al., 2013). The Preiss-Handler pathway is commonly observed to be dysfunctional in cancer cells owing to the loss of expression of nicotinic acid phosphoribosyltransferase domain containing 1(NAPRT1), which can help metabolize nicotinic acid (NA) to NAD (Watson et al., 2009; O’Brien et al., 2013; Shames et al., 2013). Because systemic NA levels are frequently insufficient to induce NAD synthesis, NAPRT1-expressing cancer cells cannot effectively utilize the NA-dependent salvage pathway (Kirkland, 2009). Tumor cells lacking NAPRT1, on the other hand, rely on NAMPT (the rate-limiting enzyme in salvage pathway) to create NAD and support cell survival, thus making them more vulnerable to NAMPT inhibitors (Olesen et al., 2010). This means modest changes in its activity can have a big impact on NAD metabolism and NAD-dependent cellular activities (Rongvaux et al., 2002; Revollo et al., 2004; Imai, 2009; Yaku et al., 2018).

NAMPT is found in all tissues of mammals, as well as lower species, such as insects, sponges, and prokaryotes, and its coding sequence is well conserved (McGlothlin et al., 2005). Nearly all of the human body’s organs—including the cytoplasm, adipose tissue, blood, liver tissue, cerebrospinal fluid, pancreatic tissue—contain NAMPT (Sun et al., 2017). The binding site and biological function of this enzyme will be explained in detail later.

**FIGURE 1:**
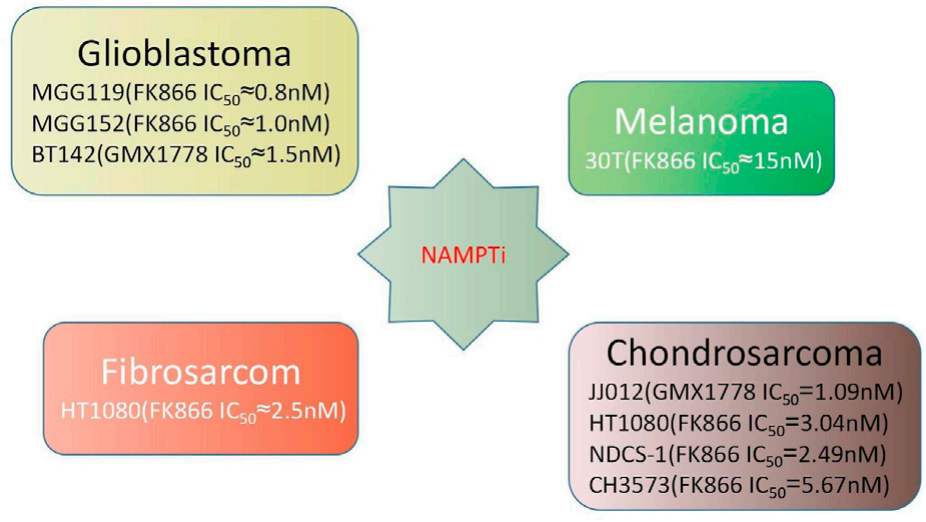
Performance of NAMPT inhibitors in various cancer cell lines (Tateishi et al., 2015; Peterse et al., 2017).

Elevated serum NAMPT levels are associated with non-alcoholic fatty liver disease, obesity, diabetes, and most significantly, malignancy (Figure 1) (Park et al., 2017; Pramono et al., 2020). In various malignancies like prostate cancer, ovarian cancer, colorectal cancer, melanoma, breast cancer, myeloma, and gastric cancer, NAMPT is commonly increased, which has an impact on the NAD pool (Reddy et al., 2008; Bi et al., 2011; Olesen et al., 2011; Maldi et al., 2013; Shackelford et al., 2013; Zhang et al., 2013; Ju et al., 2016; Lucena-Cacace et al., 2017; Hesari et al., 2018; Lee et al., 2018; Lucena-Cacace et al., 2018; Vaupel et al., 2019). NAMPT inhibitors diminish NAD levels and, as a result, hamper the cellular growth of cancer (Barraud et al., 2016; Espindola-Netto et al., 2017; Hong et al., 2019). Conversely, boosting the NAD pool has been shown to exacerbate the overexpression of NAMPT and increase the occurrence of acquired resistance to chemotherapy medicines such paclitaxel, adriamycin, fluorouracil, phenethyl isothiocyanate, and etoposide (Folgueira et al., 2005; Bi et al., 2011; Wang et al., 2011; Tome et al., 2012). In cancer cells, reducing NAMPT levels resulted in an increase in ROS and cell death. *In vitro* studies have demonstrated that employing NAMPT inhibitors to treat cancer cells, particularly cancer cell lines with IDH1/2 mutations, is considerably effective. Primary glioblastoma cell lines MGG119, MGG152,BT142, chondrosarcoma cell lines 30T, HT1080, SW1353, and gastric cancer cell lines MKN1, SNU668, Hs746T, SNU484, and SNU1750 with mutations have all been shown to be sensitive to NAMPT inhibition (Tateishi et al., 2015; Peterse et al., 2017). NAMPT inhibitors have not only showed promise as a monotherapy, but they have also been proven *in vitro* and *in vivo* investigations to prolong the therapeutic decline caused by cancer resistance to other medication treatment modalities. Such findings have lead to drug combination and dual inhibitor studies (Nahimana et al., 2009; Sampath et al., 2015; Mitchell et al., 2019). More details about the relationship between NAMPT and cancer will be described later.

On the basis of the current understanding of NAMPT regulatory mechanisms and developing evidence for NAMPT’s pathogenic functions in human cancer, the further study of NAMPT inhibitors is unquestionably significant for the treatment of cancer. However, as therapeutic demands grow, NAMPT inhibitor research and application tactics must always be adjusted. In this review, we will first present a comprehensive explanation of NAMPT’s basic biological functions and its association with cancer, then we will talk about current clinical development status and observations. Last but not the least, this review summarizes the following research and application strategies for NAMPT inhibitors: specific inhibitors, dual inhibitors, pharmacological combinations, and NAMPTi-ADCs. From a chemical point of view, We will explain the research history of each type of inhibitor, and what the different types of inhibitors learn from each other in the development process.

## 2 Binding sites for Nicotinamide phosphoribosyltransferase

**FIGURE 2:**
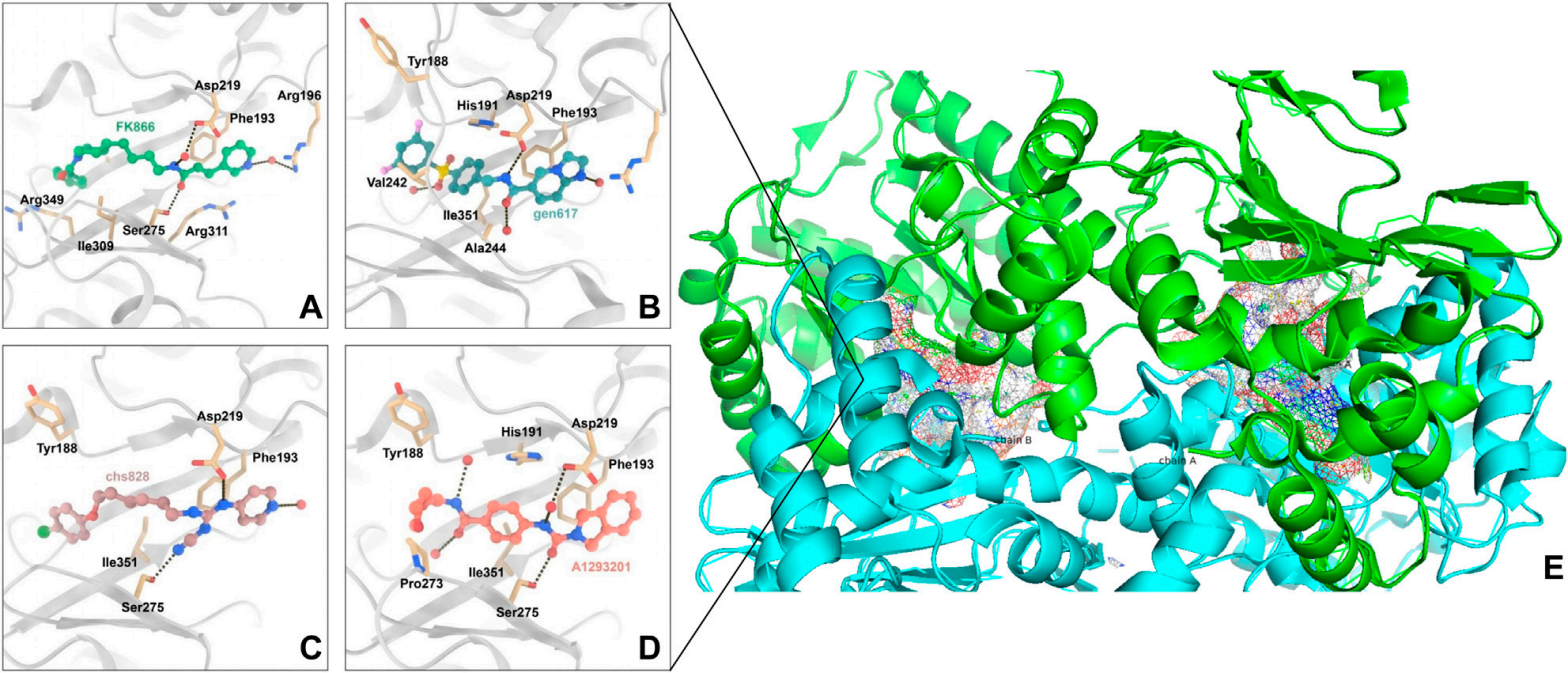
Interaction of NAMPT with inhibitory compounds: **(A)**. Interaction of NAMPT with the specific inhibitor FK866. **(B)**. Interaction of NAMPT with specific inhibitor GEN617. **(C)**. Interaction of NAMPT with specific inhibitor CHS828. **(D)**. Interaction of NAMPT with A1293201. **(E)**. X-ray crystal image of NAMPT, the enzyme has two symmetrical binding sites.

NAMPT’s enzymatic activity was first discovered by Preiss and Handler in 1957 (Preiss et al., 1958). NAMPT has a molecular weight of around 55 kDa and primarily consists of 491 amino acids. Its x-ray crystal structure has been recorded and it recognized as a dimeric class of type II phosphoribosyltransferases. NAMPT crystal structures bound to various ligands have been identified. These structures frequently contain a NAMPT homodimer with two similar active sites at the dimer interface. NAMPT inhibitors typically occupy the active site that NAM binds to, as well as a tunnel-shaped cavity which characteristically extends from an NAM binding site (Figure 2). Many NAMPT inhibitors are unique in that they rely on a nitrogen-containing heterocyclic component for cellular efficacy. When an NAMPT inhibitor binds to an NAMPT protein, the heterocyclic components protrude into the NAM binding site and mimic the covalent binding of the native substrate to PRPP (Khan et al., 2006).

Niacinamide mononucleotide (NMN) is created when NAMPT catalyzes the reversible addition of a ribosyl group from PRPP, which in terms of chemical structure is 5-phospho-D-ribosyl 1-pyrophosphate, to NAM (Garten et al., 2015). To speed up this process, NAMPT is autophosphorylated on H247, increasing the enzyme’s activity 1,125 fold and its affinity for NAM 160,000 fold (Burgos et al., 2009). The transient autophosphorylation of NAMPT does, however, strengthen the bond between the two dimeric monomers that compose the active site, increasing the enzyme’s affinity for PRPP by a factor of ten. When ATP is hydrolyzed and bound to it to create NAMN, NAMPT autophosphorylation increases catalytic efficiency 1,100 fold by lowering the Km of NAM binding from 855 to 5 nM (Burgos and Schramm, 2008). Therefore, in the presence of adequate ATP, NAMPT’s Km is 7 mM, and NAMPT is capable of efficiently converting NAM to NAMN.

## 3 Basic biological functions of Nicotinamide phosphoribosyltransferase

**FIGURE 3:**
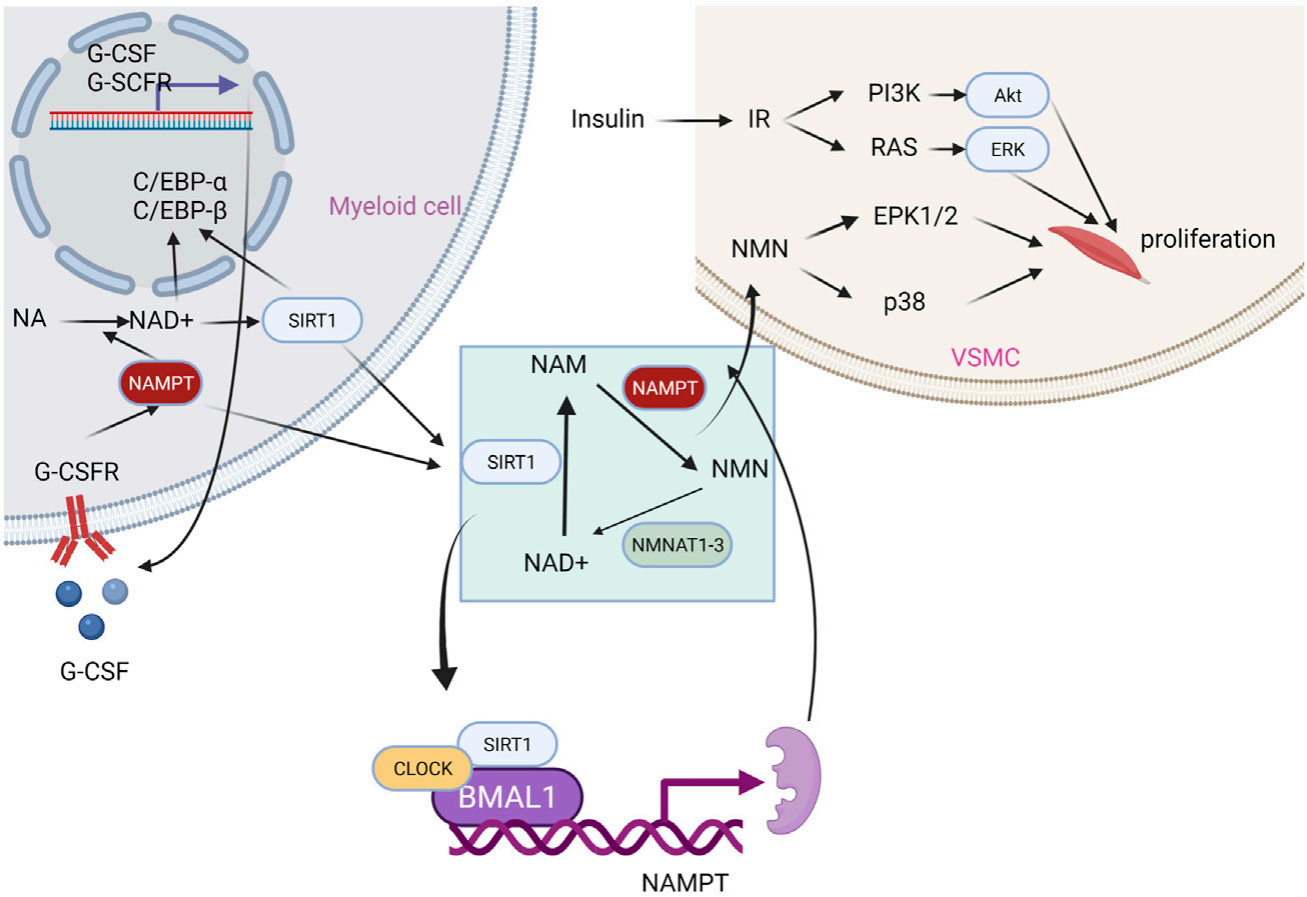
Effects of NAMPT on different physiological activities.

NAMPT has been demonstrated to play a role in the circadian clock (Figure 3). The recruitment of SIRT1 to the NAMPT promoter to boost NAMPT expression is controlled by the main component of the circadian clock mechanism. NAD production occurs next, and it triggers the activation of sirtuins and other NAD-dependent enzymes in turn. SIRT1 will suppress the component and subsequently NAMPT expression in a negative feedback loop. Through this mechanism, it is hypothesized that SIRT1 and NAMPT may be essential for metabolism circadian regulation (Nakahata et al., 2009; Ramsey et al., 2009). In the salvage pathway of NAD synthesis, its direct product, NAMN, may play a role in signaling (Wang et al., 2009; Yoshino et al., 2011). Similar to this, NAMPT can be thought of as a nicotinamide scavenger due to the fact that nicotinamide inhibits numerous NAD-utilizing enzymes.

In addition, NAMPT enhances the function of interleukin-7 and stem cell factor in the enhancement of normal human or mouse bone marrow pre-B colony formation (Dahl et al., 2012). Extracellular NAMPT functions as an immunomodulatory mediator that directly promotes inflammation in macrophages by elevating MMP production and activity; the treatment of extracellular NAMPT is dependent on MAPK signaling and induces cytokine production, monocyte chemo-taxis in human PBMCs (Moschen et al., 2007; Fan et al., 2011). In myelopoiesis, extracellular NAMPT induces granulocyte differentiation of CD34^+^ hematopoietic progenitors by activating sirtuin and upregulating G-CSF and G-CSF receptors (Skokowa et al., 2009). Finally, there is ample evidence supporting the existence of crosstalk between the extracellular NAMPT signaling pathway and the insulin signaling pathway (Fukuhara et al., 2007).

## 4 Relationship with cancer

### 4.1 Crosstalk between Nicotinamide phosphoribosyltransferase and oncogenic signaling pathways

In a number of cancer models, crosstalk between oncogenic signaling pathways and NAMPT has been documented. Some oncogenic factors have been shown to control NAMPT expression and activity, such as the oncogenic transcription factor EWS-FLI1 in Ewing’s sarcoma and the tumor suppressors FOXO1 (negatively controlling expression of NAMPT) as well as AKT (positively controlling NAMPT expression) in breast cancer (Mutz et al., 2017; Jeong et al., 2019). NAMPT can control the activation of oncogenic signaling pathways in various circumstances. For instance, extracellular NAMPT (eNAMPT) released from melanoma cells and the overexpression of NAMPT in breast cancer cells have both been linked to the activation of AKT (Grolla et al., 2015; Ge et al., 2019). Additionally, it has been discovered that exogenous eNAMPT increases the proliferation of breast cancer cells by causing the activation of AKT and ERK1/2, which can be treated with AKT and ERK1/2 inhibitors (Gholinejad et al., 2017). In multiple cancer models, NAMPT inhibition was observed to decrease phosphorylation of ERK1/2, and NAMPT inhibitors and ERK1/2 blockers together increased cell death (Cea et al., 2012; Okumura et al., 2012; Zhang et al., 2012). Numerous cancers have been linked to the interaction between mTOR and NAMPT. NAMPT inhibition resulted in the elimination of mTOR activation, and an increase in AMPKα activation in hepatocellular carcinoma cells (Schuster et al., 2015). Pancreatic ductal adenocarcinoma cells, leukemic cells, and pancreatic neuroendocrine tumor cells all displayed comparable effects, and in both subtypes of pancreatic cancer, concurrent treatment with mTOR inhibitors enhanced the antiproliferative effects of NAMPT inhibition (Zucal et al., 2015; Mpilla et al., 2019). NAMPT inhibition has also been linked in multiple myeloma models to a decrease in mTOR activation, which is assumed to be a factor in autophagic death. The alterations in NAMPT expression that take place with the emergence of resistance to targeted therapy have been the subject of numerous studies. BRAF inhibitor-resistant melanoma cells expressed more iNAMPT and eNAMPT than sensitive cells in both experimental models and clinical samples (Audrito et al., 2018a). Notably, the addition of NAMPT inhibitors can overcome resistance to BRAF inhibitors (Audrito et al., 2018b). Furthermore, inhibition of BRAF in susceptible cells results in downregulation of NAMPT transcription, whereas increase of NAMPT expression makes melanoma cells resistant to BRAF inhibitors (Ohanna et al., 2018). Based on data supporting the existence of crosstalk between NAMPT and oncogenic signaling pathways, co-targeting NAMPT with other signaling pathway molecules, such as ibrutinib, a BTK inhibitor for macroglobulinemic cells, may be a promising therapeutic strategy (Cea et al., 2016).

### 4.2 Nicotinamide phosphoribosyltransferase regulation of tumors

The expression and activity of NAMPT in malignancies are closely regulated by a number of transcriptional and post-transcriptional processes. Chowdhry and others described an NAMPT enhancer that regulates the expression and activity of NAMPT and is situated 65 kb upstream of the NAMPT transcriptional start site. Chromatin immunoprecipitation and additional research revealed that this H3K27 acetylated NAMPT enhancer binds to the transcription factors MAX and c-MYC, which control its activity, and performs substantially better exclusively in cancer cells that are dependent on the salvage pathway. An earlier work reported a c-MYC-NAMPT-SIRT1 positive feedback loop where c-MYC interacts with the NAMPT promoter and stimulates the expression of NAMPT, which in turn causes SIRT1 activation by increasing the supply of NAD. This is consistent with the involvement of c-MYC in NAMPT expression. By reducing p53 activity and blocking c-MYC-induced apoptosis, sIRT1 stabilizes c-MYC, increases its transcriptional activity, and stimulates cancer (Menssen et al., 2012). Inhibiting this loop, which is active in colorectal cancer, is thought to be a promising treatment approach (Brandl et al., 2018; Brandl et al., 2019). Another protein that is said to control the expression of NAMPT via several enhancer elements is the protein high mobility group A (HMGA1). It was shown that the HMGA1-NAMPT-NAD signaling axis drives a pro-inflammatory senescence-associated secretory phenotype (SASP) through enhancement of the activity of NF-κB, which is mediated by NAD, therefore encouraging an inflammatory environment and accelerating tumor growth (Nacarelli et al., 2019).

In fact, the overexpression of HMGA proteins in many cancer types is often associated with poor prognoses (Sumter et al., 2016). Contrarily, the tumor suppressor and transcription factor Foxo1 binds to the NAMPT gene’s 5' flanking region and reduces the expression of NAMPT in breast cancer cells, an action that is counteracted by the insulin-PI3K-AKT signaling pathway. In addition, expression of NAMPT in triple-negative breast cancer (TNBC) is epigenetically regulated by a new promoter-associated Lnc-RNA, NAMPT-AS “RP11-22N19.2”. At both transcriptional and post-transcriptional stages, NAMPT-AS stimulates expression of NAMPT, which drives the tumor development process and enhances the aggressiveness of TNBC (Zhang et al., 2019). Another long-stranded non-coding RNA, gastric cancer-associated transcript 3 (GACAT3), promotes the development of gliomas by functioning as a molecular sponge for miR-135a, blocking its interaction with NAMPT, and controlling NAMPT production (Wang et al., 2019). According to various studies, microRNAs control NAMPT expression at the post-transcriptional level. Specifically, NAMPT mRNA is the target of miR-26b in colorectal cancer, miR-23b in melanoma, miR-381, miR-206, miR-494 and miR-154 in breast cancer cells, and miR-206 in pancreatic cancer (Bolandghamat Pour et al., 2019a; Bolandghamat Pour et al., 2019b; Ghorbanhosseini et al., 2019; Lv et al., 2020). In general, increased expression of these microRNAs can inhibit NAMPT expression and is associated with a decrease in cancer cell viability, suggesting that these microRNAs may have potential as anticancer drugs. From this discovery we know that the activity of NAMPT enzymes can be controlled by other enzymes in addition to being regulated at the gene level. Some research reported that SIRT6 specifically boosts NAMPT’s enzymatic activity through direct protein deacetylation to shield cancer cells from oxidative stress (Sociali et al., 2019). Similar to this, a prior study discovered that SIRT1 deacetylates NAMPT, making it easy for adipocytes to secrete (Yoon et al., 2015). Through the transcription factor C/EBP-β, mesenchymal glioblastoma stem cells selectively increase the expression of NAMPT and NNMT and interact with their gene regulatory areas. Notably, in these cell subtypes, NNMT downregulated DNA methyltransferase expression in a methionine-dependent way and generated a DNA hypomethylation condition (Jung et al., 2017).

### 4.3 Current clinical development status and observations

Following encouraging findings in preclinical research, the therapeutic safety and efficacy of NAMPTis have been assessed in people with cancer. There are ten NAMPT inhibitor trials registered on clinicaltrials.gov as of January 2022 (Hjarnaa et al., 1999; Jonsson et al., 2000; Ekelund et al., 2001). The first human NAMPT inhibitor trial (NCT00003979) began in 1999 but was halted in 2012 due to serious side effects and poor clinical outcomes. Among the initial NAMPTis trials were phase I studies using CHS828, which is an NAMPT inhibitor which was developed in the early years of research on such compounds for solid tumors. They established the required dose of CHS828, but nevertheless, future trials were not recommended due to illness progression and major side events experienced by the participants in the study (Hovstadius et al., 2002a; Ravaud et al., 2005; Von Heideman et al., 2010). A clinical trial in Phase II for cutaneous T-cell lymphoma utilizing the NAMPT inhibitor FK866 was halted due to the failure to establish remission and the occurrence of substantial side effects (Goldinger et al., 2016). These poor outcomes were attributed to target off tumour toxicity as well as cellular uptake of NAD sources, including nicotinic acid riboside, vitamin B3, and tryptophan (Table 1) (Grozio et al., 2013). More details are provided in the NAMPT Inhibitors section.

**TABLE 1:** Clinical trials on NAMPT inhibitors (updated to January 2022).

Drug	Phase	Conditions	Status	Nut identifier	Outcome measures	Display of results on ClinicalTrials.gov
GMX1777	Ⅰ	Solid Tumors and Lymphomas	Withdrawn	NCT00457574		No Results Posted
GMX1777+, Temozolomide	ⅠⅡ	Metastatic Melanoma	Terminated	NCT00724841	Primary Outcome Measures: Determine the recommended Phase II dose of GMX1777 in combination with temozolomide [ Time Frame: 2 years ] Learn more about the side effects of taking GMX1777 in combination with temozolomide [ Time Frame: Within the first 4 weeks ] Determine the disease response to treatment with GMX1777 in combination with temozolomide [ Time Frame: Within the first 8 weeks ]	No Results Posted
					Secondary Outcome Measures: Learn more about how the body processes GMX1777 [ Time Frame: Within the fisrt 30 days ]	
CHS828	Ⅰ	Unspecified Adult Solid Tumor, Protocol Specific	Withdrawn	NCT00003979		No Results Posted
APO866	Ⅰ Ⅱ	B-cell Chronic Lymphocytic Leukemia	Completed	NCT00435084	Primary Outcome Measures: Safety and tolerability of APO866 in patients with refractory B-CLL not amenable to allogeneic HSCT [ Time Frame: 1 month]	
					Secondary Outcome Measures: To determine the effect on the number of circulating leukemic after treatment as compared to baseline [Time Frame: 1 month]. To determine the effect on the number of CD38^+^ after treatment as compared to baseline [Time Frame: 1 month]	
					Correlative analysis on *in vivo* and *in vitro* sensitivity of leukemic cells, CD38 expression of leukemic cells and clinical outcome, immunophenotype and clinical outcome [Time Frame: 1 month]	
APO866	Ⅱ	Melanoma	Completed	NCT00432107	Primary Outcome Measures: To determine the tumor response rate (according to Response Evaluation Criteria in Solid Tumors (RECIST) criteria) as the proportion of eligible patients with stage IV cutaneous melanoma or stage III not amenable to surgery. [Time Frame: Week 16]	No Results Posted
					Secondary Outcome Measures: Safety and tolerability [Time Frame: Week 16 and 12 months follow-up]	
					Time to response [Time Frame: Week 16]	
					Duration of response [Time Frame: Week 16]	
					Progression free survival [Time Frame: 12 months]	
					Overall survival [Time Frame: 12 months]	
					Evolution of serum VEGF and interleukin-8 (IL-8) during treatment [Time Frame: Week 16]	
APO866	Ⅱ	Cutaneous T-cell Lymphoma	Completed	NCT00431912	Primary Outcome Measures: The proportion of eligible patients with refractory or relapsed CTCL whom have a complete response or partial response on cutaneous lesions (Tumor Burden Index) and extra-cutaneous disease. [Time Frame: Week 16]	No Results Posted
					Secondary Outcome Measures: Safety and tolerability, time to response, duration of overall response, duration of stable disease and time to treatment failure. [Time Frame: Week 16]	
KPT-9274	Ⅰ	Acute Myeloid Leukemia Acute Myeloid Leukemia, in Relapse Acute Myeloid Leukemia Refractory	Recruiting	NCT04914845		No Results Posted
KPT-9274	Ⅰ	Solid Tumors NHL	Terminated	NCT02702492	Primary Outcome Measures: Maximum tolerated dose (MTD) for KPT-9274 administered alone and with co-administration of niacin ER (extended release) (vitamin B3/nicotinic acid) [Time Frame: Approximately 4 weeks]	No Results Posted
KPT-9274&Niacin					Parts A and B: MTD will be based on the assessment of dose limiting toxicities (DLTs) during the first cycle of therapy and will be defined as the highest dose at which ≤1 participant out of 6 (or 0 out of 3) experiences DLTs within Cycle 1	
ER					Maximum tolerated dose (MTD) for KPT-9274 co-administered with nivolumab [ Time Frame: Approximately 4 weeks]	
KPT-9274 + Nivolumab					Part C: MTD will be based on the assessment of dose limiting toxicities (DLTs) during the first cycle of therapy and will be defined as the highest dose at which ≤1 participant out of 6 (or 0 out of 3) experiences DLTs within Cycle 1	
ATG-019	Ⅰ	Solid Tumor	Recruiting	NCT04281420		No Results Posted
ATG-019 + Niacin ER		Non-Hodgkin’s Lymphoma				
OT-82 Dose Escalation	Ⅰ	Lymphoma	Recruiting	NCT03921879		No Results Posted
OT-82 Dose Expansion		Lymphoma, Non-Hodgkin				
		Lymphoma, B-Cell (and 4 more)				

## 5 Nicotinamide phosphoribosyltransferase inhibitors

Using NAMPT as the target for oncology drugs, compounds with better inhibitory effects such as FK866, CHS828, GEN617, OT-82 and other cytotoxic compounds have been developed over the years. The development of NAMPT inhibitors with better therapeutic efficacy and dosing strategies will continue to be explored. This review will summarize the current research already conducted on NAMPT inhibitors, focusing upon specific inhibitors, drug combinations, dual inhibitors and NAMPTi-ADCs.

### 5.1 Specific inhibitors

#### 5.1.1 FK866

**FIGURE 4:**
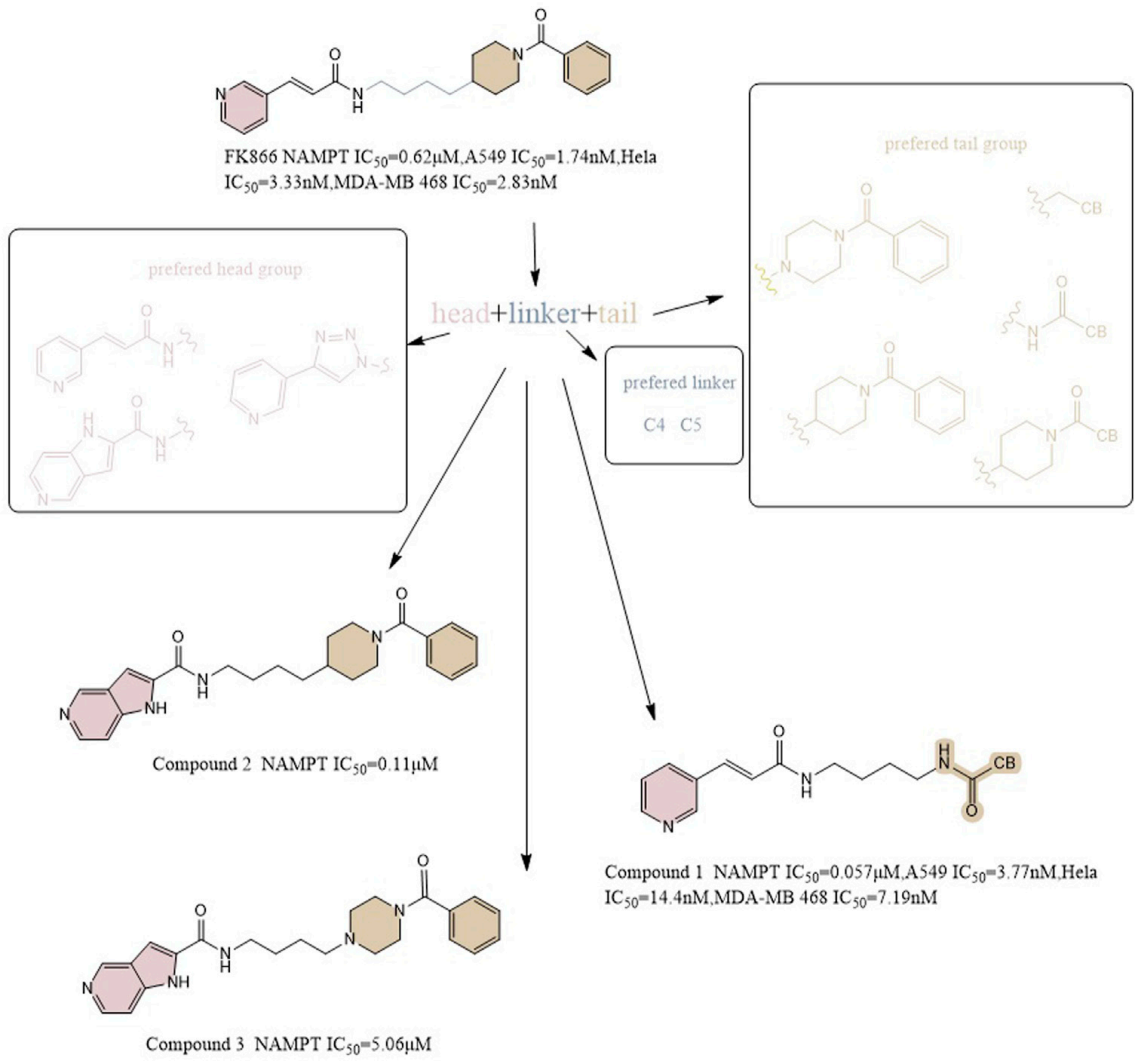
Structural optimization of NAMPT inhibitors based on FK866 as a prototype.

Max Hasmann and Isabel Schemainda identified in 2002 a novel class of compounds with a characteristic induction of delayed cell death by high-throughput screening, by which (E)-N-[4-(1-benzoylpiperidin-4-yl) butyl]-3-(pyridin-3-yl) acrylamide (FK866) became the first reported NAMPT inhibitor, which could be a candidate anticancer drug with an IC_50_ of about 1 nM and which indirectly suppresses mitochondrial respiratory activity, but selectively inhibits NAPRT, causing gradual NAD depletion (Hasmann and Schemainda, 2003a). NAMPT is a homodimer with two unique tunnel-shaped cavities adjacent to each active site. Crystallographic studies of the complex of FK866 and NAMPT revealed that FK866 binds to the tunnel cavity of NAMPT. The researchers divided the structural components of this family of NAMPT inhibitors into head (an aromatic moiety similar to pyridine), linker (a tunnel-interacting moiety), and tail components (a solvent-exposed group) based on the structure of FK866 (Figure 4) (Asawa et al., 2019). Subsequently, Sei-ichi Tanuma et al. performed substitutions for the head and tail groups and finally found that the head group with pyridinylacrylamide, the intermediate linker with a 4-carbon chain, and the tail group with p-carborane induced the best inhibitory activity, which was 10 times greater than that of FK866 (IC_50_ ∼1 nM). The hydrogen bonding interactions between carborane amide and His191 can be used to explain these findings (Figure 2A) (Asawa et al., 2019; Tanuma et al., 2020).

#### 5.1.2 CHS828 and GMX1777

**FIGURE 5:**
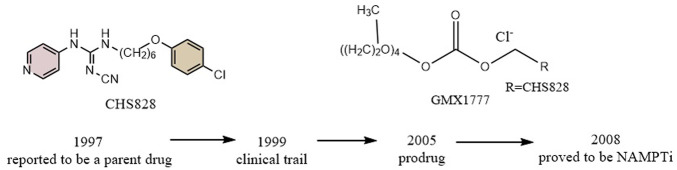
Discovery and research history of the NAMPT inhibitor CHS828.

CHS828, a pyridinyl cyanoguanidine anticancer drug which was developed by Leo PharmaAS, whose chemical structure can also be divided into a head group, a linker and a tail group. It was reported upon by Schou and his colleagues in 1997. It showed strong anticancer activity in lung and breast cancer cell lines. In experiments with nude mice, treatment with CHS828 led to the regression of human lung and breast cancer tumors (Schou et al., 1997; Hjarnaa et al., 1999). CHS828 was examined in a clinical trail in 1999 (Hovstadius et al., 2002b), but it wasn’t until 2008 that Olesen’s research demonstrated that the compound can block NAMPT and kill cancer cells primarily by way of depleting NAD (Olesen et al., 2008). While attempting to address the solubility and pharmacokinetic issues which had been noted in the early clinical trials for CHS828, Ernst et al. created a number of prodrugs during this time that had improved properties. The best of these compounds was GMX1777, whose tetraethylene glycol portion was attached to the parent drug via a carbonate bond (Figure 5). Its solubility allows it to be rapidly released *in vivo* through intravenous injection. It exerts very strong inhibitory activity *in vivo* when used in combination with a cytostatic agent etoposide (Binderup et al., 2005). Crystallographic analysis revealed that CHS828, like FK866, exerts its activity by binding to the NAMPT tunneling cavity (Figure 2C).

#### 5.1.3 GEN617 and LSN3154567

**FIGURE 6:**
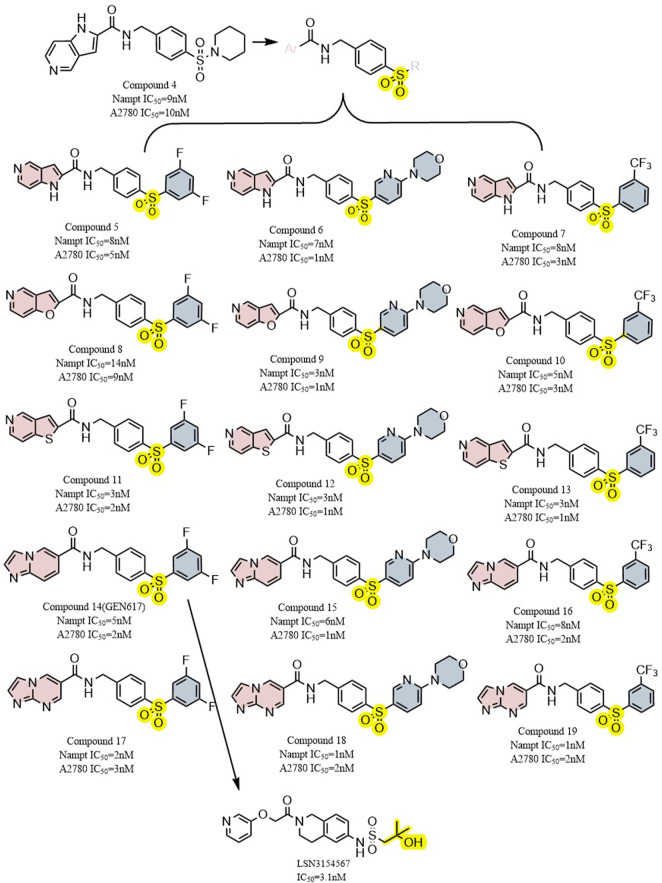
Structural modification of sulfur-containing NAMPT inhibitors and the typical compound GEN617,LSN3154567.

Through structure-guided design and the use of significant data from the eutectic structure of (thio) urea, Zheng et al. found a structurally unique NAMPT inhibitor which contains amide. Further optimization led to GEN617(IC_50_ < 10 nM vs. MiaPaCa2, PC3, HT1080, U251, and HCT116 lines), which has good *in vitro* and *in vivo* ADME qualities and single-digit nanomolar cellular semi-inhibitory doses against a number of human cancer cell lines, guided by the crystal structure of compound4 (NAMPT IC_50_ = 9 nM and A2780 IC_50_ = 10 nM) with NAMPT complexes. In crystallographic analysis, Asp219 and the amide NH were observed to form a hydrogen link, and Ser275 and the amide carbon were seen to form a water-mediated hydrogen bond. In the NAM binding area, GEN617’s bicyclic imidazolopyridine ring is stacked between Tyr18 and Phe193 of the NAMPT residues, and the imidazole nitrogen points to the PRPP binding site (Figure 2B) (Zheng et al., 2013). However, GEN617 carries the risk of serious side effects, such as retina damage. NAMPT inhibitors’ capacity to traverse the blood-retinal barrier is diminished by altering their polarity and permeability, which lowers the retina’s exposure to the drugs. Using these findings for as a basis for further research, Genshi Zhao discovered a unique NAMPT inhibitor, LSN3154567 (IC_50_ = 3.1 nM), which changed the ring of the tail group part to a hydrocarbon group, and did not cause retinal lesions in rats (Figure 6). The examined dogs’ retinal and hematologic toxicity was fully removed by co-administrating NA without any appreciable reductions in effectiveness (Zhao et al., 2017).

#### 5.1.4 A1293201

**FIGURE 7:**
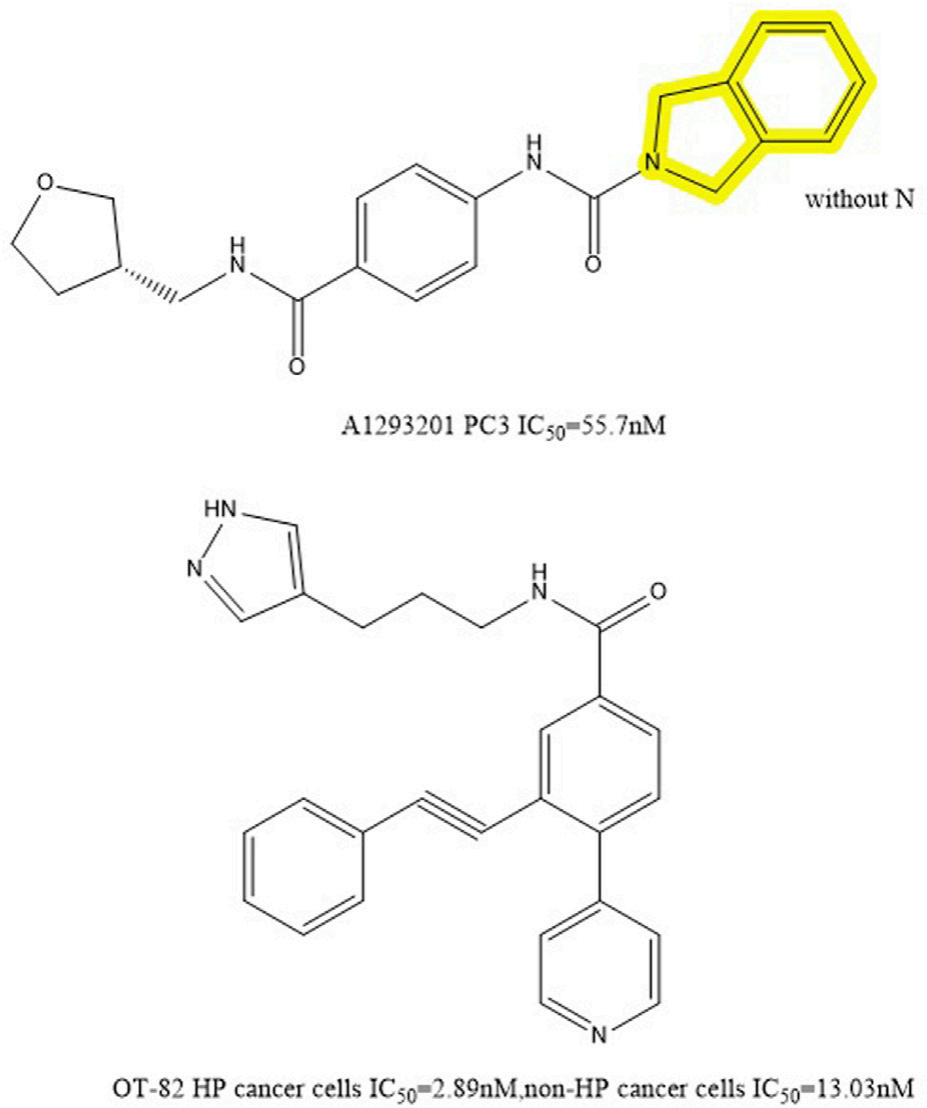
Chemical structure and half inhibition concentration of A1293201 and OT-82.

Through high-throughput cell screening and target identification, Julie et al. identified a series of novel non-substrate NAMPT inhibitors with robust preclinical efficacy and pharmacokinetic properties for oral administration. The candidate molecule in this series, A1293201 (PC3 IC_50_ = 55.7 nM), contains a heteroenopeptide “headgroup” that does not have the aromatic nitrogen of nicotinamide and nicotinamide-like compounds compared to previously identified inhibitors, such as FK866, GMX1777 and GNE617 (Figure 7). Crystallographic studies have shown that similar to FK866 and nicotinamide, A1293201 binds to the nicotinamide site and engages in crucial pi-stacking interactions with tyrosine 18; the central region of the compound passes through a narrow lipophilic tunnel to reach the distal opening of the active site (Figure 2D) (Wilsbacher et al., 2017).

According to Julie et al., the conventional wisdom that this property is necessary for cellular efficiency is challenged by powerful and selective NAMPT inhibitors like A1293201 which lack aromatic nitrogen in the nicotinamide moiety. Despite the lack of phosphorylation sites, the efficacy of A1293201 and other isoindoles may lead to intracellular retention or other unexpected effects. Therefore, A1293201 or other isoindolines may help regulate NAMPT inhibition in normal cells more effectively.

#### 5.1.5 OT-82

The new NAMPT inhibitor OT-82 was created by OncoTaris and began Phase I clinical trials in 2019. A high-throughput cell-based screen of a chemical library of over 200,000 small molecules led to the discovery of this lead compound, which was then validated and structurally improved (Figure 7). It inhibits NAMPT through NAD and ATP depletion and induces apoptotic cell death. It demonstrates higher action against hematopoietic malignancies (IC_50_ = 2.89 ± 0.47 nM) than against non-hematopoietic tumors (13.03 ± 2.94 nM) (Wilsbacher et al., 2017). OT-82 did not exhibit the same neurological, cardiac, or retinal toxicity as other NAMPTis in toxicological investigations in mice and non-human primates. The principal targets of OT-82 for dose-limiting toxicity in both species were found to be the lymphoid and hematopoietic organs.

#### 5.2 Drug combinations

**FIGURE 8:**
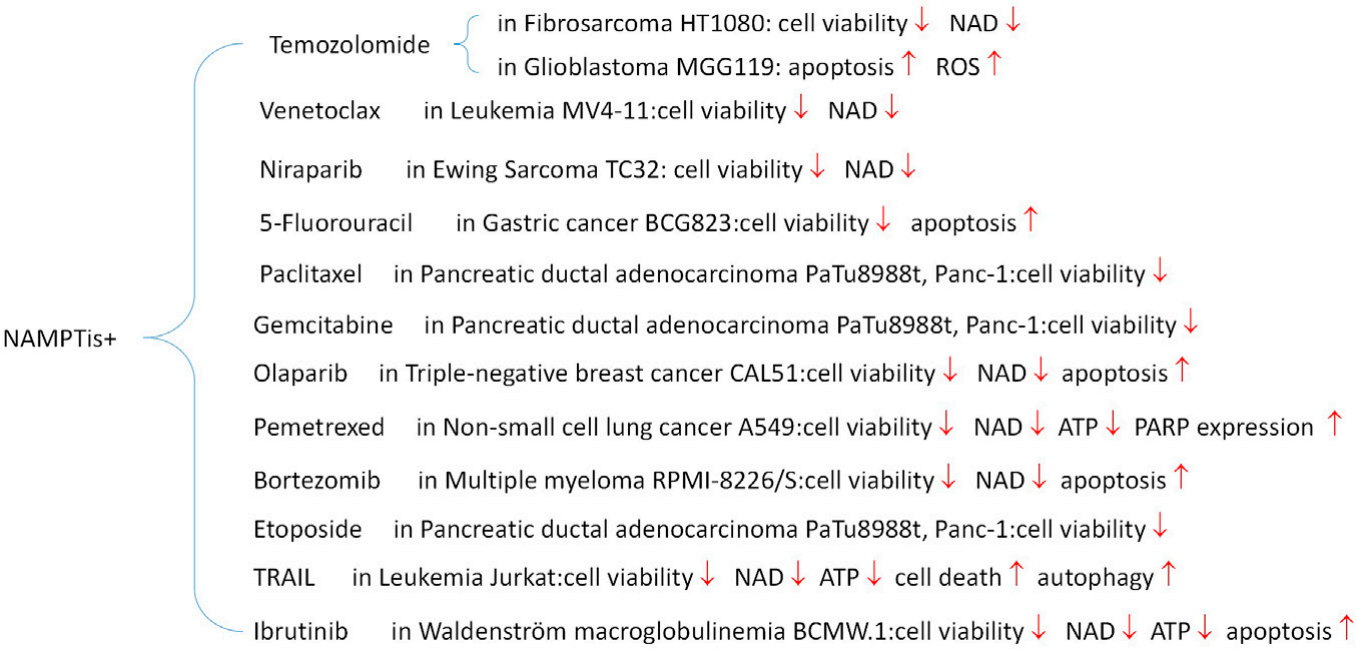
Performance of drug combination *in vitro*.

Although specific inhibitors have shown good cytotoxicity and targeting *in vivo* and *in vitro* experiments, they often face problems such as large toxic side effects in clinical trials, including the aforementioned neurological, cardiac and retinal toxicity. Additionally, single-target inhibitors become less effective over time with the development of drug resistance. Moreover, cancer is a multifactorial disease, so the application of only a single specific inhibitor has some inherent limitations. Although there is no clear literature or clinical data that the application of combinations of drugs carries a lower risk of toxic side effects than using a single specific inhibitor, it has been documented that this strategy does have the potential to exert synergistic effects, enhance therapeutic effects, as well as delay and reduce the development of drug resistance (Figure 8).

#### 5.2.1 PARPi + NAMPTi

**FIGURE 9:**
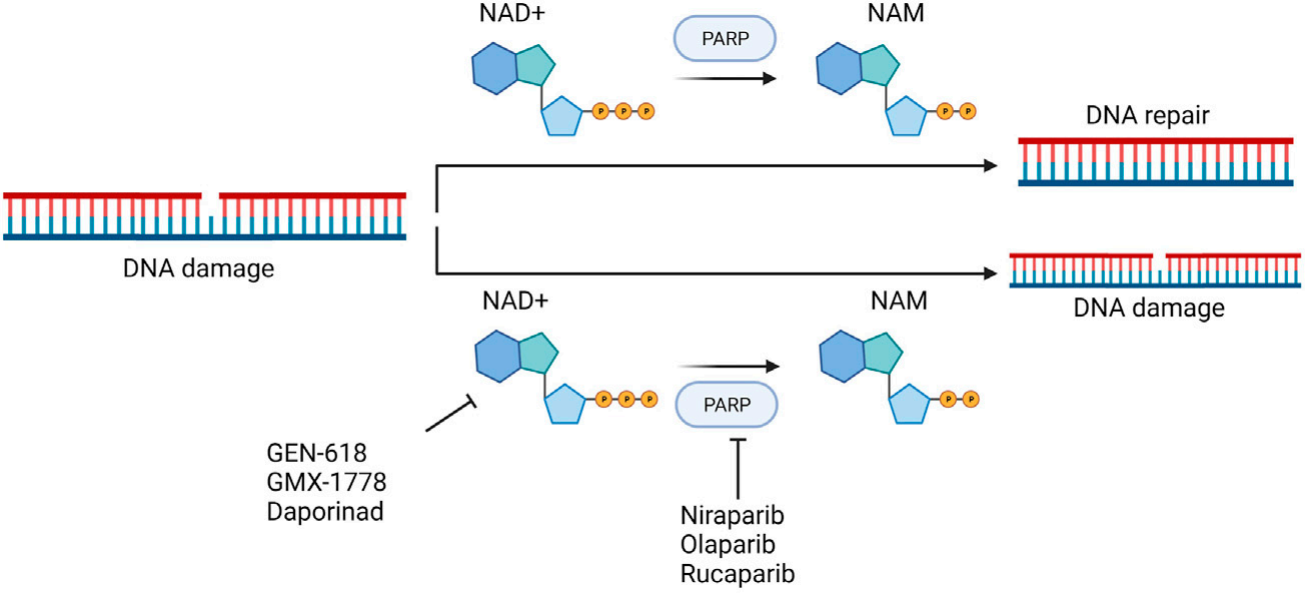
Theoretical basis of simultaneous targeting of PARP and NAMPT in tumor therapy.

Combinatorial drug matrix screening is an efficient method of identifying new therapies which show synergistic potential *in vitro*. Christine et al. performed drug combination screening for Ewing’s sarcoma and identified several potentially effective combinations of drugs, including PARPis and NAMPTis (Heske et al., 2017). Using a multi-omics approach, this study also found that the apoptotic phenotypes and DNA damage which resulted from the combination were associated with NAD+ and NMN loss, strengthened PARP suppression, and persistent activation of cellular stress pathways (Figure 9).

Currently there is an abundance of preclinical evidence which supports the theory that Ewing’s sarcoma gene fusions depend on PARP1 activity and that tumor cell lines harboring these fusions are extremely vulnerable to PARP blocking (Brenner et al., 2012). Unfortunately, a xenograft model of Ewing’s sarcoma cannot duplicate the single-agent efficacy of PARP inhibitors (Ordonez et al., 2015; Smith et al., 2015). The addition of NAMPTis to PARPis is a potential combination therapy which would theoretically enable use of lower PARP inhibitors dosages by further reducing PARP activity, which may simultaneously make the therapy both more clinically effective and less toxic. Because the known toxicity profiles of both inhibitors seem to differ from one another, this kind of combination may be more tolerable for patients. In addition to Ewing’s sarcoma, triple-negative breast cancer xenograft models have demonstrated that the combined inhibition of PARP and NAMPT slows tumor growth to some extent. Even in cases in which treatment has been delayed until the tumor becomes very large, this combined treatment still often results in tumor regression (Bajrami et al., 2012).

The study by Christine et al. may provide a new and complementary account of the mechanisms of this type of drug combination: the stress-activated protein kinases p38 MAPK and SAPK/JNK were co-activated by NAMPT and PARP inhibition, and PAR activity was considerably decreased both *in vitro* and *in vivo* following NAMPT and PARP inhibition (Heske et al., 2017).

#### 5.2.2 NAMPTi + NAPRTi

Departing slightly from the previous combination, we might also consider another important pathway of NAD + production in order to identify and eliminate more NAMPT inhibitor resistance mechanisms. For this purpose we may shift our focus to another important target, nicotinic acid phosphoribosyltransferase (NAPRT). This is a second enzyme which produces NAD + and mediates the nicotinic acid (NA) to NAD + conversion. In a number of prevalent malignancies, the gene encoding is amplified and overexpressed, including ovarian cancer, where NAPRT expression correlates with the BRCAness gene expression profile.

Hara and his coworkers demonstrated that NAPRT is required for NA to raise cellular NAD + levels in human cells, that NAPRT and NAMPT both do the same for intracellular NAD + levels, as well as guard against the toxicity of hydrogen peroxide (Hara et al., 2007). In addtion, Francesco’s study on NAPRT shows that it plays previously undiscovered yet significant roles, demonstrating that in cancer cells which overexpress NAPRT, the compound also helps to maintain intracellular NAD + pools under basal conditions, and that, in a subpopulation of tumors, NAPRT-dependent NAD + biogenesis aids DNA repair processes and tumor cellular metabolism. Francesco and his colleagues performed a series of validation experiments that yielded several intriguing results: 1) Cancer cells overexpressing NAPRT were insensitive to FK866. 2) NAPRT reduced DNA damage in cancer cells and regulated cytoplasmic and mitochondrial NAD + levels, oxidative phosphorylation and protein synthesis. 3) In cancer cells that overexpress NAPRT, inhibition of NAPRT by 2-hydroxinicotinic acid reduces OXPHOS and makes them sensitive to FK866. 4) NAPRT inhibition or silencing could lead to sensitization of ovarian cancer xenografts to FK866. These findings strongly suggest that 1) NAPRT genes are amplified and overexpressed in a subset of common cancers; 2) NAPRT plays a role in cancer cells’ (e.g., ovarian and pancreatic cancer cells) energy status, cell growth, metabolism, DNA repair processes, proliferation regulation and protein synthesis; 3) Resistance to NAMPT inhibitors in previously tried clinical trials may be overcome by NAPRT inhibition (Piacente et al., 2017).

#### 5.2.3 VB3/other targeted agents + NAMPTi

Early studies on NAMPTis showed that exogenous addition of niacin (vitamin B3) could rescue cellular NAD + depletion and cytotoxicity via the PreissHandler pathway (Watson et al., 2009). This method depends only on the expression of NAPRT1 (Xiao et al., 2013). Because NAPRT1 is expressed in most mammalian tissues, this combination is generally an option for patients with NAPRT1-negative tumors. Combining NAMPTi’s with niacin allows it to meet both clinical efficacy and safety requirements. Studies in mice have shown that when niacin is administered in combination with doses of NAMPTi which would otherwise be lethal, it can reduce the occurrence of problems related to mortality and toxicity, such as thrombocytopenia and lymphopenia. Using this tactic, several of the toxicities problems seen in human clinical trials were lessened. Coadministration with nicotinic acid significantly decreased histological evidence of toxicity to spleen, testicular, and lymphoid tissues and reversed damage to less afflicted kidney, liver, and gastrointestinal tissues (O’Brien et al., 2013).

In addition, NAMPTi was found to be effective in the following combinations: with Vorinostat for CLL and acute myeloid leukemia, with TRAIL for t-cell leukemia and CLL, with FX-11 for LDHA-dependent lymphomas, with Bortezomib for multiple myeloma, and with Rituxumab for B-cell lymphoma (Le et al., 2010; Zoppoli et al., 2010; Cea et al., 2011; Cagnetta et al., 2013; Nahimana et al., 2014).

We have found that combined drug therapy is more toxic to tumor cells not only in theory, but also *in vivo* and *in vitro* experiments, and that it can also solve the problem of drug resistance to some extent. However, the pharmacokinetic problems of two drugs in clinical trials are more demanding for modeling and difficult to control in practical applications.

#### 5.3 Dual inhibitors

Although the strategy of drug combination solves to some degree the problems of drug resistance and greater toxicities that exist when treating with single drugs, the pharmacokinetic problems derived from the simultaneous use of two drugs are challenging to overcome in clinical practice. We want to take advantage of the efficacy of drug combinations and solve pharmacokinetic problems. Synthesizing hybrid molecules that can display two different balanced mechanisms of action, i.e., a dual inhibitor, is a desirable strategy that can be adopted in drug development. Drugs with polypharmacological activities have now been shown to be more beneficial than combination therapy because they often have fewer severe side effects and provide doctors with a more flexible treatment approach.

#### 5.3.1 PAK4-Nicotinamide phosphoribosyltransferase dual inhibition

A known β-catenin protein regulator, p21-activated kinase 4(PAK4), a RhoGTPase(CDC42) effector, has been proven to control WNT signaling. In the cytoplasm, p21-activated kinase four phosphorylates β-catenin on Ser675, preventing its degradation and boosting its transcriptional activity. The changed cell polarity observed in several malignancies is caused by p21-activated kinase 4, which co-localizes with β-catenin at the cell membrane in the cell junctional region, phosphorylating it. Downstream effects of PAK4 inhibition include a reduction in G2-M transition, and downregulation of nuclear β-catenin, c-MYC and CyclinD1. In addition, inhibition of NAMPT resulted in significant depletion of NAD and downregulation of SIRT1 activity.

**FIGURE 10:**
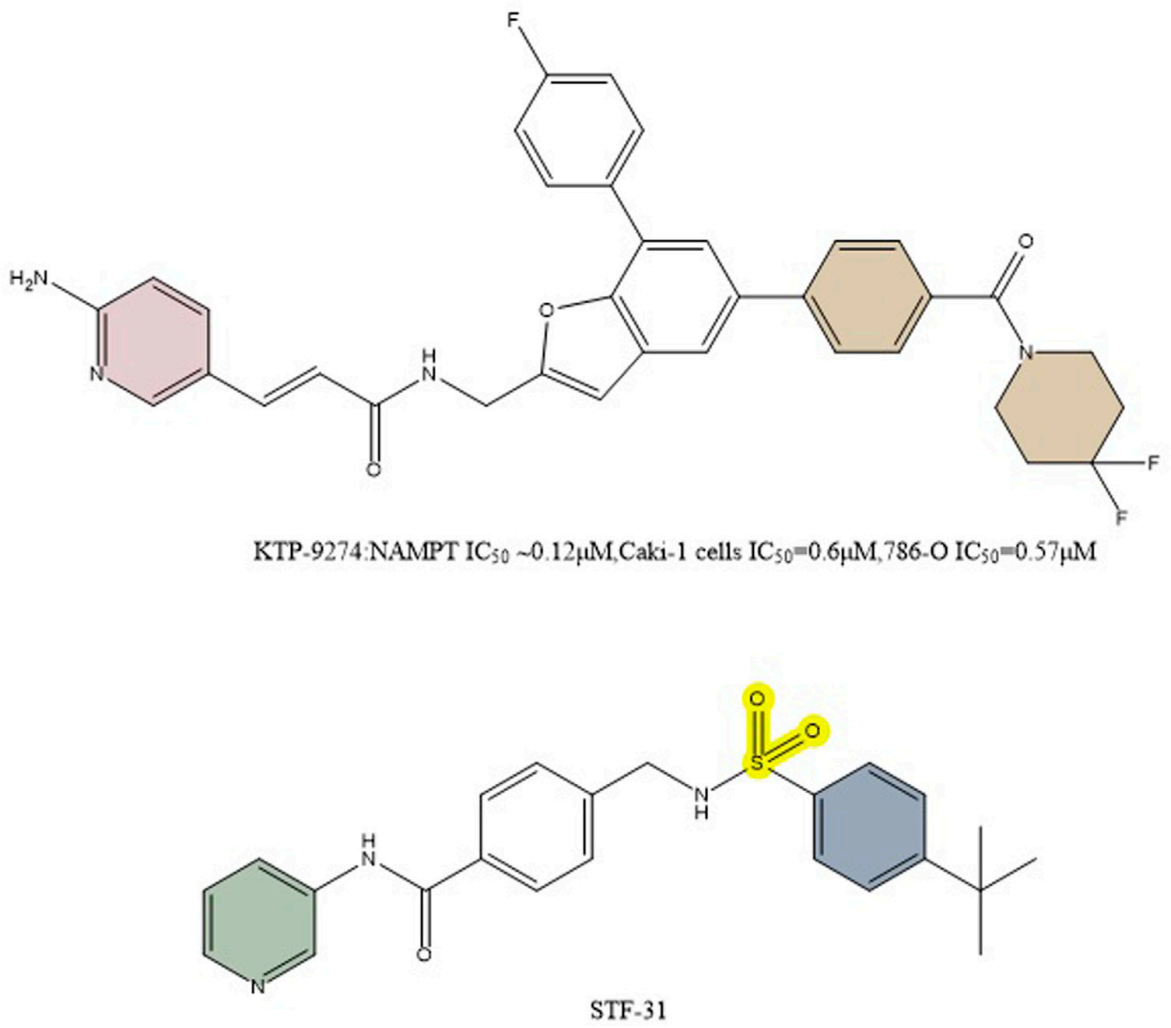
Structure of dual inhibitors KPT-9274 and STF-31.

Based on preclinical evidence, it has been determined that combined inhibition of NAMPT and PAK4 may be a successful anticancer tactic. A broad-spectrum PAK inhibitor called PF-3758309 blocks both class A PAKs (PAKs 1, 2, and 3) and class B PAKs (PAKs4,5,6) (Murray et al., 2010). It was discovered that FK866 is a very effective inhibitor of NAMPT (Hasmann and Schemainda, 2003b). Despite strong preclinical data for both compounds, both inhibitors were discontinued due to a lack of objective responses in phase I studies. In preclinical studies, the dual PAK4-NAMPT inhibitor KPT-9274 (NAMPT IC_50_ ∼0.12 μM,Caki-1 cells IC_50_ = 0.6 μM,786-O IC_50_ = 0.57 μM) exhibited strong effectiveness against a variety of solid tumors and hematologic malignancies in both *in vivo* and *in vitro* settings (Figure 10) (Abu Aboud et al., 2016; Jiang et al., 2016; Aboukameel et al., 2017; Fulciniti et al., 2017; Rane et al., 2017). KPT-9274 has minimal cytotoxicity to normal primary kidney cells and when used in mice no significant weight loss was observed. The only known drug that targets both NAMPT and PAK4 is KPT-9274, which is the subject of NCT02702492 listed in Table 1. In this clinical trial, Gabriel et al. concluded that KPT-9274 could make pancreatic neuroendocrine tumor cells more sensitive to traditional mTOR inhibitors, resulting in better therapeutic outcomes (Mpilla et al., 2021). KPT-9274 has been demonstrated to modulate Wnt/β-linked protein signaling and decrease the growth of multiple tumor cell lines and subcutaneous xenograft models of mantle cell lymphoma. Non-Hodgkin’s lymphoma cell lines are efficiently subjected to KPT-9274’s dose-dependent induction of apoptosis (Azmi et al., 2017). KPT-9274 was well tolerated in subcutaneous non-Hodgkin’s lymphoma xenografts in mice and exhibited significant antitumor activity.

#### 5.3.2 GLUT1-Nicotinamide phosphoribosyltransferase dual inhibition

Currently, tumor therapy is thought to be made possible by the transfer of glucose during glycolysis and its metabolism (Porporato et al., 2011). Two distinct transporter molecules, the facilitated glucose transporter (GLUT), which facilitates energy-independent bidirectional transport, and the Na^+^/glucose cotransporter (SGLT), which transports glucose actively, are involved in the uptake of glucose by cells. There are 13 structurally related members of the GLUT family in humans, and GLUT1 is the one that is most frequently expressed in primary tissues and cultured cells (Zhao and Keating, 2007). The predominant subtype of the glucose transporter that is overexpressed in cancer is GLUT1 (Moreno-Sánchez et al., 2007). After ingestion, glucose is metabolized to triphosphate via the glycolytic pathway, following additional oxidation. Trisaccharide-phosphate oxidation via glyceraldehyde 3-phosphate dehydrogenase reduces NAD^+^, which is needed for this step as an electron acceptor in the glycolytic pathway, to NADH. Without NAD + regeneration, glycolysis cannot be sustained. By converting pyruvate to lactate, which subsequently oxidizes NADH to NAD^+^, which serves as an electron acceptor, this regeneration is accomplished.

STF-31’s impact on NAD + metabolism and glucose transport was examined by Kraus et al. in comparison to GLUT inhibitors and NAMPT inhibitors. Unlike other GLUT mono-inhibitors, STF-31 exhibited some tumor suppression even in the low concentration range (below 5 μM), especially for the osteosarcoma MG-63 (Figure 10). Both STF-31 and GLUT inhibitors inhibited glucose uptake in tumor cells. Like the NAMPT monoinhibitors, STF-31 also significantly reduced cell viability. The findings suggest that STF-31 has a dual function and that the inhibitory effect is concentration-dependent, so a number of cancer cells studied appear to be more sensitive to its lethal effects as a given NAMPT inhibitor. Each inhibitory activity may have a different impact depending on the particular cellular setting. Cells with low expression levels of NAMPT may respond better to the drug’s inhibitory impact on NAMPT while cells with high expression levels of GLUT1 may be more sensitive to the drug’s inhibitory effect on GLUT1 when they also have low expression levels of other GLUTs (Kraus et al., 2018).

#### 5.3.3 HDAC-Nicotinamide phosphoribosyltransferase dual inhibition

The regulation of gene expression is greatly influenced by the post-translational alterations of chromatin histones. Histone acetyltransferases (HATs) and histone deacetylases (HDACs), two of the most extensively researched epigenetic changes, regulate balanced acetylation/deacetylation of lysines in the core histone tails. A regulated equilibrium between histone acetylation and deacetylation appears to be necessary for optimal cell proliferation (Yang and Seto, 2007). Many disorders, including neurodegenerative diseases, inflammatory diseases, and cancer, are thought to be influenced by the dysregulation of histone deacetylases (Oppermann, 2013). For example, in neuroblastoma, elevated expression of HDAC8 is linked to advanced illness and poor chances of survival. (Oehme et al., 2009); In patients with ovarian and gastric cancer, elevated expression levels of HDAC1, 2, and three have been linked to poor prognoses (Weichert et al., 2008a; Weichert et al., 2008b).

**FIGURE 11:**
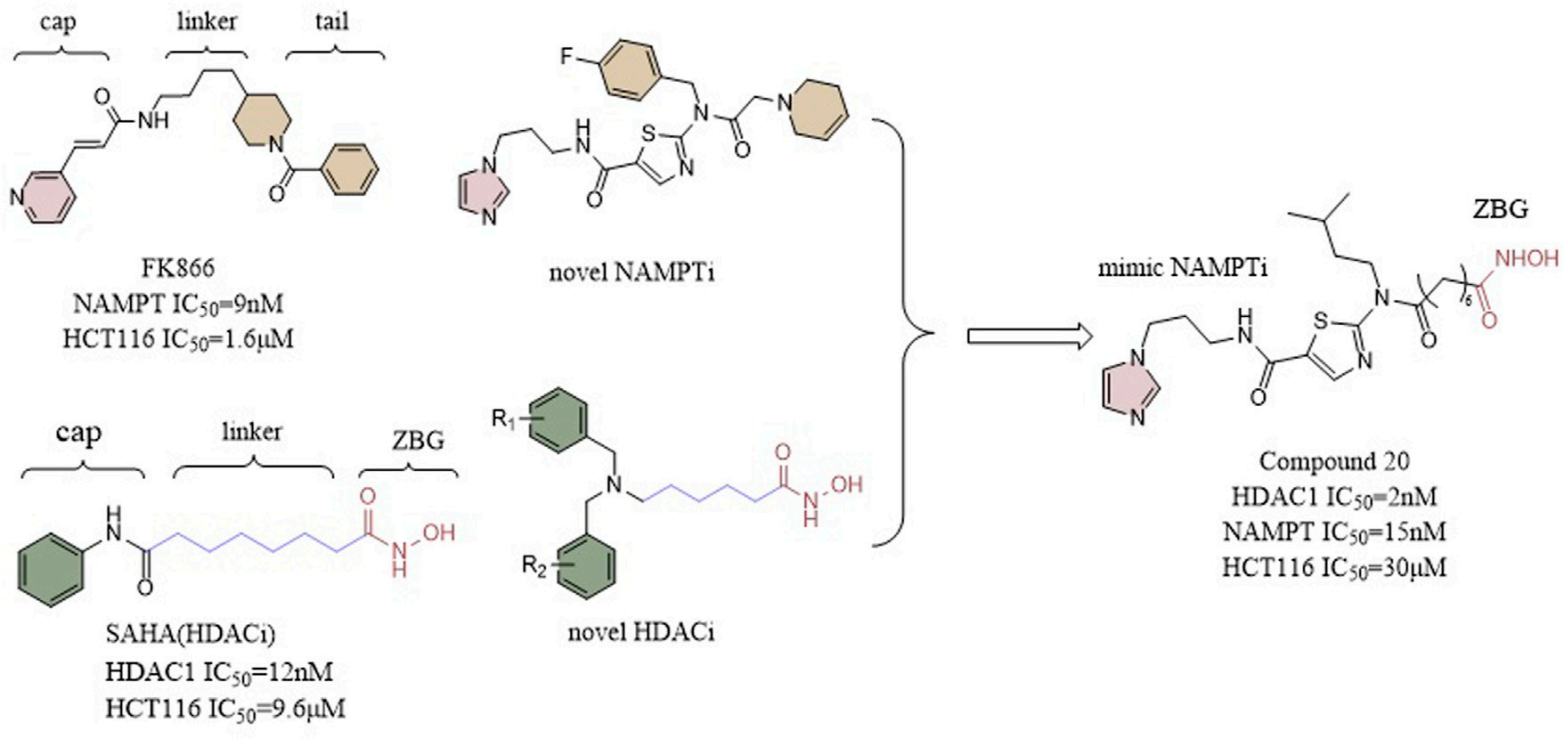
Structure design of HDAC and NAMPT dual inhibitors in Chen’s research.

It has been demonstrated that NAMPT inhibitors increase the inhibitory effect of HDAC inhibitors, indicating that compounds with dual inhibition abilities may present potent antitumor activity. In a study by Chen et al. a potent inhibitor of NAMPT was bound to a zinc hydroxamic acid binding group via a 6-carbon alkyl linker creating compound 20 (IC_50_ = 2 nM), which, in nanomolar range, demonstrated a better inhibitory effect against HDAC-1 than Vorinostat 1(IC_50_ = 12 nM). Furthermore, compound 20 exhibited a considerably stronger inhibitory effect on NAMPT in comparison to FK866. *In vitro* anti-proliferative assays showed that compound 20 was no less potent than Vorinostat one and standard FK866 against the colon cancer HCT116 cell line. In vivo assays, the anti-tumor activity exhibited by compound 20 (TGI = 42%, T⁄C = 58%) was also higher than that of SAHA (T⁄C = 67%, TGI = 33%) and FK866 (T⁄C = 61%, TGI = 39%) (Figure 11) (Chen et al., 2018).

**FIGURE 12:**
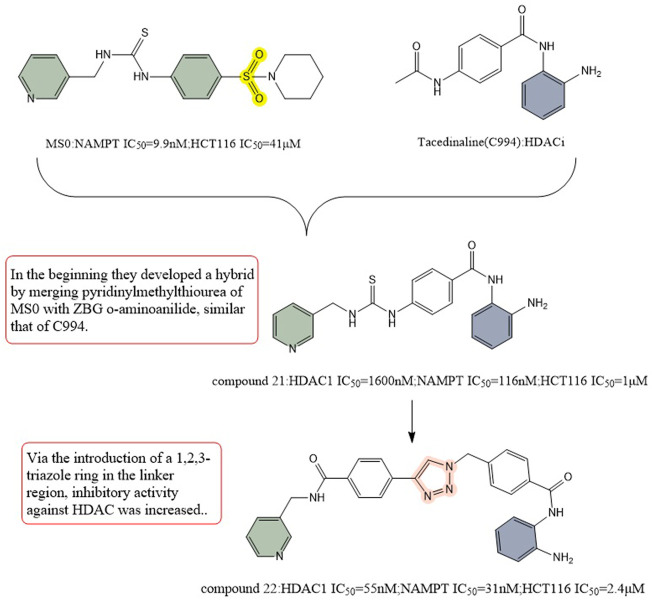
Structure design of HDAC and NAMPT dual inhibitors in Dong’s research.

Dong et al. (2017) then replaced ZBG hydroxymethyl ester with 2-aminobenzamide, and their first designed dual inhibitor was prepared by linking MS0 (NAMPT inhibitor) with Tacedinaline (C994, HDAC inhibitor). Compared to MS0 and Tacedinaline, compound 21 exhibited weaker enzyme inhibition of NAMPT and HDAC1. To achieve balanced activity against both enzymes and improve the enzymatic efficiency, they created additional structural alterations. The HDAC inhibition activity was increased by introducing a 1,2,3-triazole ring in the linkage region, resulting in compound 22 (IC_50_ = 1.6 μM), which exhibited better HDAC inhibition activity than Tacedinaline. In addition, compound 22 showed stronger NAMPT inhibitory activity compared to MS0. Compound 22 showed reduced antiproliferative activity against HCT-116 cell line cancer compared to compound 21. The TGI of compound 22 at the same dose (TGI = 69%) was far more higher than that of standard SAHA (TGI = 33%) and FK866 (TGI = 39%). These results make it a potential precursor for further structure-activity-relationship studies with the aim of introducing more promising HDAC/NAMPT hybrid inhibitors (Figure 12).

#### 5.4 NAMPTi-antibody-drug conjugate

Efficacious medicinal medications are delivered by antibody-drug conjugates (ADC) to the targeted diseased spot while protecting healthy tissue from adverse side effects (Chari, 2016). Because problems related to targeting and dosage toxicity limit the clinical usage of NAMPT inhibitors, such as thrombocytopenia and gastrointestinal effects, targeted delivery to tumor tissues via the ADC pathway may significantly improve the therapeutic index. This method is expected to improve the therapeutic window for NAMPT inhibition.

**FIGURE 13:**
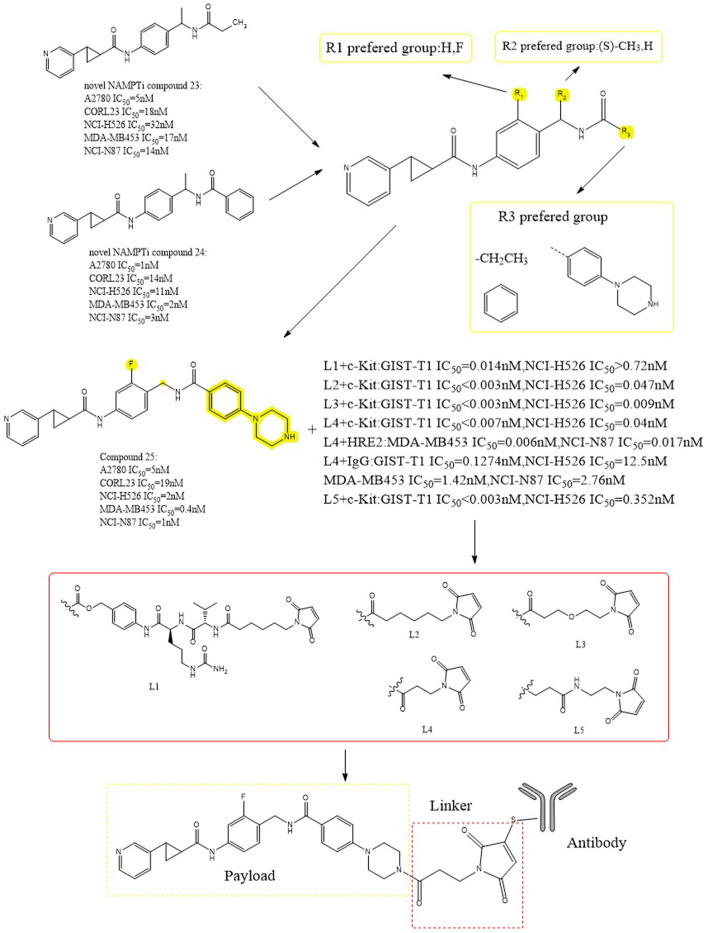
Structural design of NAMPTi-ADCs.

Alexei et al. carried out a series of structural modifications using compounds 23 and 24 as lead payloads to detect their biological activities (Figure 13) (Palacios et al., 2018). Among them, compound 25 showed better tumor cytotoxicity as a payload (A2780 IC_50_ = 5 nM), which demonstrated greater inhibitory activity against the following cells: NCI-H526 (high expression of c-kit antigen, IC_50_ = 2 nM), MDA-MB453 (high expression of HER2 antigen, IC_50_ = 0.4 nM) and NCI-N87 (high expression of HER2 antigen, IC_50_ = 1 nM). Five different cleavable and noncleavable linkers were selected to be paired with c-kit, HER2, and IgG antibodies. The experimental results showed that L4 was the better linker. When targeting c-kit and when targeting HER2, data showed a lower IC_50_ (for the targeting of c-kit, the IC_50_ in NCI- H526, was 0.04nM; for HER2, IC_50_ was 0.006 nM in MDA-MB453 and 0.017 nM in NCI-N87) (Karpov et al., 2018). These conjugates showed *in vivo* effectiveness that was target-dependent while being well tolerated.

**FIGURE 14:**
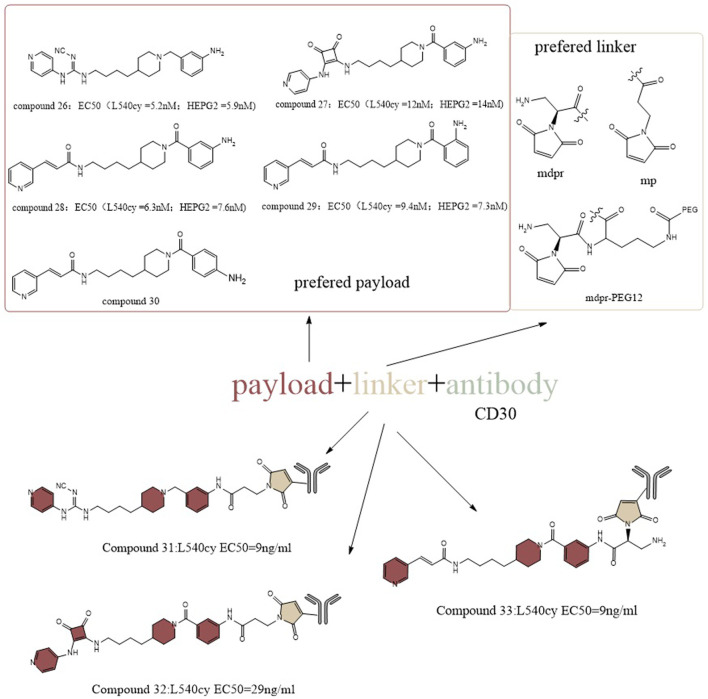
Structural design of NAMPTi-ADCs.

In another study, Christopher et al. designed the payload by structurally guided modification of the prototype inhibitor FK866 (Figure 14) (Neumann et al., 2018). They installed an amino group to the benzene ring of the tail group portion of FK866 and applied a pyridyl-cyanoguanidine group and a pyridylsquaramide group from the structure of CHS-828 in the head group portion to generate a new toxic payload, which retained cytotoxicity in cells. Among them, compound 26 has a lower EC50 (∼5.2 nM for L540cy and ∼5.9 nM for HEPG2) and greater cytotoxicity than FK866 (∼6.7 nM for L540cy and ∼6.8 nM for HEPG2). To increase the molecule’s hydrophilicity on the linker side, a glucoside-based linker system was created. For the antibody side, CD19, CD30 and CD123 were chosen as targets for the design. The final result was a payload and a linker which worked together to give ADCs outstanding biophysical characteristics. From the activity point of view, they showed antigen-specific NAD depletion activity *in vitro*. After 96 h of treatment, ATP depletion was weak or absent in some cell lines, likely due to the kinetics of the drug release and internalization of the ADC. To achieve optimal action, rapid and persistent drug exposure could be required. As a result, the long circulation half-life of ADC *in vivo* may provide ongoing drug exposure to antigen-positive target cells, making ADC administration an ideal method of NAMPT suppression. The *in vivo* anticancer efficacy of this group of ADC have been proven in numerous xenograft models of acute myeloid leukemia, non-Hodgkin’s lymphoma (Burkittlymphoma and follicular lymphoma, CD19 as antigen, 60%–100% of mice with tumor regression), and Hodgkin’s lymphoma (CD30 as antigen, 80% of mice with tumor regression when administered at 10 mg/kg) (Neumann et al., 2018).

## 6 Conclusion

Cellular metabolism and signaling are affected by the production of NAD, the utilization of NAD as an ADPR donor, and the maintenance of NADPH homeostasis. Therefore, it is not surprising that activity within these pathways is frequently greater in patients with cancer. NAD-producing enzyme presents a set of targets that can be used to combat the proliferation of cancer. The salvage pathway is the main source of human NAD and is dependent on NAMPT, the rate-limiting enzyme in this cyclic pathway. Small alterations to its bio-activity can have a big impact on NAD-dependent cellular processes and NAD metabolism. Of the three NAD-producing pathways, it is currently the only one through which effective cancer therapy can be administered. Beginning with the study of CHS828 in 1997, we have seen many creative monomeric NAMPT inhibitors, such as GMX1777, CHS828, OT-82, and more. Lead compounds for small molecule NAMPT inhibitors are often obtained by fragment-based screening, virtual screening, and high-throughput screening. Even though NAMPT is the most frequently employed NAD-producing enzyme in chemical inhibitor applications, the low therapeutic efficacy and high toxicity of NAMPT inhibitors in clinical trials have slowed down the development of new such drugs. Cancer is a multifactorial disease, so manipulation of only a single target may fail to produce the desired to therapeutic outcomes. Therefore, increasing the clinical availability of NAMPT inhibitors requires more drug designs and innovative drug delivery strategies. Drug combinations can, to some extent, reduce drug toxicity and side effects, exploit drug synergy and enhance therapeutic effects, as well as delay and reduce the development of drug resistance, but there are inherent pharmacokinetic problems when using two different drugs simultaneously that must be overcome. Therefore, using a single drug that influences two or more targets may be a more effective strategy. Currently, combination therapies have been shown to be more beneficial than drugs that exhibit polypharmacological activity because they have lower side effects and allow for more flexible treatment approaches. Dual-target-single-drug strategies have become a popular approach to cancer treatment, and more and more dual-target drugs which can target NAMPT are being developed. Currently the most common of these strategies include targeting PAK4 and NAMPT together, GLUT1 and NAMPT together, and HDAC and NAMPT together. Dual inhibitors, may in theory, solve to some extent the problems of drug resistance, high toxicity and pharmacokinetic coordination of the two classes of drugs, but the actual efficacy of such therapeutic strategies is still yet to be seen in clinical trials. Thus, there is still a need for the development of new effective inhibitors based on new pairs of targets. Targeted delivery of cytotoxic payloads to tumor tissues via the ADC pathway may significantly improve therapeutic indices and is expected to improve the therapeutic window for NAMPT inhibition. In addition to determining the *in vivo* activity of such ADC payloads, meaningful advances to this type of therapy can only continue to pursue clinical applications if toxicity profiles are improved relative to the systemic administration of NAMPT inhibitors. This review briefly summarizes the practical application and experimental validation of the strategies using specific inhibitors, drug combinations, dual inhibitors, and ADCs, with the intention of aiding and inspiring future research on NAMPT-targeted oncology drugs. The further development and design of novel, selective, highly efficient, low toxicity and low molecular weight NAMPT inhibitors will not only help basic pathology research, but also bring great hope for the clinical treatment of tumors involving NAMPT to the benefit of more patients.
